# Associations between Physical Activity, Sedentary Time, and Locomotive Syndrome Differ by Age and Sex: A Cross-sectional Study

**DOI:** 10.1298/ptr.E10330

**Published:** 2025-04-07

**Authors:** Wataru NAKANO, Etsuko OZAKI, Michitaka KATO, Satoko NAKANO, Kazuya KITO, Teruhide KOYAMA

**Affiliations:** 1 Department of Shizuoka Physical Therapy, Faculty of Health Science, Tokoha University, Japan; 2 Department of Epidemiology for Community Health and Medicine, Kyoto Prefectural University of Medicine, Japan; 3 Department of Rehabilitation, Shizuoka Medical Center, Japan

**Keywords:** Locomotive syndrome, Physical activity, Physical activity intensity, Sedentary time, Sex differences

## Abstract

Objectives: Physical activity is a relevant factor for the locomotive syndrome (LS); however, the association between intensity of physical activity and LS is unclear. This study aimed to investigate the associations among different intensities of physical activity (vigorous, moderate, and light), sedentary time, and LS. Methods: A cross-sectional analysis of records from 2890 Japanese community residents (mean age: 57.5 years) was conducted. LS was assessed using the stand-up test, two-step test, and 25-question Geriatric Locomotive Function Scale. Physical activity and sedentary time were assessed by self-administered questionnaires. The associations among physical activity, sedentary time, and LS were examined by logistic regression analysis stratified by age (<65 years and ≥65 years) and sex. Results: Vigorous physical activity in men aged ≥65 years, and moderate and vigorous physical activities in women aged <65 years were significantly associated with lower odds of LS. By contrast, no significant association was found between physical activity and LS in men <65 years and women ≥65 years of age. No association between light physical activity and the LS was found in either group. The association between sedentary time and LS was observed in women aged <65 years. Conclusions: Physical activity and sedentary time were associated with the LS but in an age- and sex-dependent manner. This study highlights the importance of engaging individuals in high-intensity physical activity to prevent or address LS.

## Introduction

Aging is linked to chronic diseases and disabilities, emphasizing the importance of maintaining independent living in the context of an aging global population^1)^. The Japanese Orthopaedic Association introduced the concept of locomotive syndrome (LS) in 2007. LS is characterized as a condition marked by diminished mobility due to impaired locomotive organs, thereby increasing the risk of disability^2,3)^. Identifying the factors associated with LS is essential as it guides interventions aimed at preventing LS and subsequent disability.

Physical activity and exercise habits^4–6)^ are well-established factors related to LS, with sedentary time also suggested as a contributing factor^7)^. However, several unresolved issues exist regarding the relationships among physical activity, sedentary time, and LS. First, the relationship between physical activity intensity and LS remains unclear. While previous studies have reported the association between the presence and frequency of exercise habits and LS^4–6)^, research on the intensity of physical activity is lacking. Determining this association is important because higher-intensity physical activity has been shown to reduce the incidence of frailty and functional disability compared to lower-intensity activity^8,9)^. Second, the consistency in the relationships among physical activity, sedentary time, and LS is uncertain across age groups and sexes. Studies suggest a varying association between exercise frequency and LS across age groups^6)^. Additionally, the association between physical activity and LS varies by sex^10)^. Stratified analysis by age and sex may help identify those who would benefit the most from intensive promotion of physical activity for LS prevention. Although a limited number of studies have reported an association between sedentary time and LS^7)^, further research is required to gain more evidence on this association.

Therefore, this study aimed to investigate the association among various intensities of physical activity, sedentary time, and LS across different age groups and sexes.

## Methods

### Study participants

This study used data from the Kyoto field of the Japan Multi-Institutional Collaborative Cohort (J-MICC) study. The J-MICC study is a cohort study that aims to examine gene-environment interactions in lifestyle-related diseases, especially cancer^11,12)^. The J-MICC study consists of multiple research sites across Japan, including the Kyoto field. In the Kyoto field of the J-MICC study, a baseline survey was conducted between 2008 and 2012 among people aged 35–69 years, followed by a follow-up survey between 2013 and 2017. This study used data from the follow-up survey, focusing on individuals who were 40–75 years of age at the time of the follow-up survey and had no missing data required for analysis, resulting in a dataset of 2890 participants. All participants provided written informed consent for participation, and ethical approval was obtained from the Institutional Ethics Committee of the Kyoto Prefectural University of Medicine (ERB-E-36). This study was conducted in accordance with the principles of the World Medical Association’s Declaration of Helsinki.

### Assessment of LS

LS was assessed using the stand-up test, two-step test, and 25-question Geriatric Locomotive Function Scale (GLFS-25)^3,13,14)^. In the stand-up test, the participants were instructed to stand up with a single leg or both legs from platforms of different heights (40, 30, 20, and 10 cm). The difficulty levels were as follows: standing up with both legs from platforms of 40, 30, 20, and 10 cm in height, followed by standing up with a single leg from platforms of the same height. In the two-step test, the participants were instructed to take two steps from a standing position without losing balance. The two-step score was calculated by dividing the length of the two steps by the participant’s height. The GLFS-25 is a self-administered questionnaire comprising 25 questions covering the past month. It encompasses four dimensions: four questions on pain, 16 on activities of daily living, three on social functioning, and two on mental health. Each question is rated on a scale of 0–4, with higher scores indicating more severe symptoms. Based on the results of these three tests, LS is classified into four stages: non-LS and LS-1 to LS-3. In this study, LS-1 to LS-3 were categorized as with LS. Specifically, individuals were classified as LS if they met any of the following criteria: inability to stand on one leg from a 40 cm platform during the stand-up test, a score of <1.3 on the two-step test, or a score of ≥7 on the GLFS-25.

### Evaluation of physical activity levels and sedentary time

Leisure-time physical activity and daily-life physical activity were assessed using self-administered questionnaires^15–17)^. To assess leisure-time physical activity levels, we used a questionnaire similar to the International Physical Activity Questionnaire (IPAQ). Participants were required to report the frequency and average duration of three physical activity intensities: vigorous, moderate, and light. Vigorous activities were those that made participants panting and unable to talk; moderate activities were those that made participants panting but still able to talk; and light activities were those that did not make participants panting. The vigorous, moderate, and light activities were assigned 8, 4, and 3.3 metabolic equivalents (METs), respectively. The frequency categories (assigned average days per week) for leisure-time physical activity were as follows: almost none (0), one to three times per month (0.1), one to two times per week (0.2), three to four times per week (0.5), and five to six times per week (0.8). The average duration categories (assigned average hours) were as follows: <30 minutes (0.3), 30 minutes to <1 hour (0.8), 1 to <2 hours (1.5), 2 to <3 hours (2.5), 3 to <4 hours (3.5), and ≥4 hours (4.5). The activity level (METs-hours/day) for each activity was determined by multiplying the METs by the frequency and duration of the activity. In addition, total leisure-time physical activity level was defined as the sum of the activity levels of the three categories.

For daily-life physical activity, participants reported the duration of hard labor, walking, and standing per day by choosing from eight possible responses (assigned average hours): none (0), <1 hour (0.5), 1 to <3 hours (2), 3 to <5 hours (4), 5 to <7 hours (6), 7 to <9 hours (8), 9 to <11 hours (10), and ≥11 hours (11). Participants were also asked to report their average hours of sleep per day. Physical activity levels for hard labor and walking per day were calculated by multiplying the activity intensity (assigned 4.5 METs for hard labor and 3.3 METs for walking) by the duration of the activity. In addition, the total daily-life physical activity level was calculated by summing the activity levels of the two categories. Daily sedentary time was calculated by subtracting the sum of hard labor, walking, standing, and sleeping from 24 hours^17)^.

### Clinical parameters

The data on the clinical parameters used in this study were obtained from personal health records. Hypertension was characterized by the use of antihypertensive medications, systolic blood pressure of ≥140 mmHg, or diastolic blood pressure of ≥90 mmHg. Diabetes mellitus was defined as the use of oral hypoglycemic drugs or a fasting blood sugar level of ≥126 mg/dL. Hyperlipidemia was defined as the use of lipid-lowering medications, a total cholesterol level of ≥220 mg/dL, or a low-density lipoprotein cholesterol level of ≥140 mg/dL.

### Statistical analysis

Data are expressed as mean ± standard deviation for continuous variables and as frequency and percentage for categorical variables. Group differences in numerical variables were assessed using Welch’s t-test, whereas frequency differences were assessed using the chi-squared test. The associations among physical activity, sedentary time, and LS were examined using logistic regression analysis. Leisure-time physical activity was used as an explanatory variable, as previous studies have reported an association between exercise habits and LS^4–6)^. Daily-life physical activity was included in the model as an adjustment variable to account for physical activity in daily life. Logistic analyses, stratified by age (<65 years and ≥65 years) and sex, were conducted to investigate whether the associations between physical activity, sedentary time, and LS remained consistent across different age groups and sexes. Statistical analyses were performed using SPSS Statistics (version 29.0; IBM, Armonk, NY, USA). Statistical significance was set at P <0.05.

## Results

The average age of the participants was 57.5 ± 10.0 years, and 1052 (36.4%) were men. Table 1 shows a comparison of the characteristics of participants with and without LS. Of the 1052 men, 500 (47.5%) had LS. Compared to men without LS, those with LS were older and had higher body mass index (BMI) and prevalence of hypertension and diabetes. Total leisure-time physical activity levels did not differ according to LS status. However, men with LS engaged in higher levels of light physical activity, whereas those without LS participated in more vigorous physical activities. Among the 1838 women, 1014 (55.2%) had LS. Compared with those without LS, women with LS were older and had higher BMI and prevalence of hypertension and hyperlipidemia. While the total leisure-time physical activity between women with and without LS did not differ, vigorous physical activity was higher among women without LS. No difference was observed in the daily-life physical activity and sedentary time between those with and without LS, regardless of sex.

**Table 1.** Comparison of characteristics of participants with and without locomotive syndrome

	Men (n = 1052)			Women (n = 1838)		
	Non-LS	LS	P	Non-LS	LS	P
	(n = 552)	(n = 500)		(n = 824)	(n = 1014)	
Age (y)	55.8 ± 9.9	62.0 ± 9.4	<0.001	53.8 ± 9.2	59.1 ± 9.7	<0.001
BMI (kg/m^2^)	23.3 ± 2.6	23.6 ± 3.2	0.043	21.0 ± 2.8	21.9 ± 3.5	<0.001
Alcohol consumption, n (%)	416 (75.4)	374 (74.8)	0.833	433 (52.5)	483 (47.6)	0.036
Smoking, n (%)	88 (15.9)	98 (19.6)	0.120	31 (3.8)	51 (5.0)	0.191
Hypertension, n (%)	244 (44.2)	281 (56.2)	<0.001	192 (23.3)	359 (35.4)	<0.001
Hyperlipidemia, n (%)	252 (45.7)	257 (51.4)	0.062	443 (53.8)	676 (66.7)	<0.001
Diabetes mellitus, n (%)	28 (5.1)	59 (11.8)	<0.001	15 (1.8)	33 (3.3)	0.055
Total physical activity (METs-hours/day)	13.2 ± 10.3	12.8 ± 9.2	0.537	13.2 ± 8.7	13.6 ± 9.4	0.386
Leisure-time physical activity (METs-hours/day)						
Total	3.0 ± 3.8	3.0 ± 3.8	0.881	2.3 ± 3.2	2.2 ± 3.2	0.321
Light	1.3 ± 1.9	1.7 ± 2.3	0.008	1.2 ± 1.7	1.3 ± 1.8	0.170
Moderate	1.2 ± 2.3	1.2 ± 2.6	0.684	0.9 ± 2.1	0.8 ± 2.1	0.136
Vigorous	0.5 ± 1.6	0.2 ± 1.1	0.003	0.2 ± 0.9	0.1 ± 0.7	0.004
Daily-life physical activity (METs-hours/day)						
Total	10.2 ± 8.7	9.8 ± 7.5	0.417	10.9 ± 7.4	11.4 ± 8.1	0.157
Hard labor	3.9 ± 7.2	3.3 ± 5.3	0.136	3.3 ± 5.2	3.7 ± 5.8	0.100
Walking	6.3 ± 4.4	6.4 ± 4.7	0.543	7.7 ± 5.2	7.8 ± 5.2	0.697
Sedentary time (hours/day)	11.6 ± 3.7	11.4 ± 3.5	0.515	9.7 ± 3.2	9.9 ± 3.3	0.149

Values are mean ± standard deviation or frequency (%).

Locomotive syndrome was assessed using the stand-up test, two-step test, and 25-question Geriatric Locomotive Function Scale.

LS, locomotive syndrome; BMI, body mass index; METs, metabolic equivalents

Table 2 shows the results of logistic regression analysis for the presence of LS. In the total population and sex-specific subgroups, a higher leisure-time physical activity was associated with lower odds of LS (model 1). Furthermore, a consistent trend toward lower odds of LS with a higher intensity of physical activity was observed (model 2). However, statistical significance was only observed for moderate and vigorous physical activities in the total population and for moderate and vigorous physical activities in women. Sedentary time showed an association with LS in women.

**Table 2.** Association between leisure-time physical activity, sedentary time, and locomotive syndrome

	Total (n = 2890)		Men (n = 1052)		Women (n = 1838)	
	OR (95% CI)	P	OR (95% CI)	P	OR (95% CI)	P
Model 1						
Total	0.96 (0.93, 0.98)	<0.001	0.97 (0.93, 1.00)	0.057	0.95 (0.92, 0.98)	0.002
Sedentary time	1.03 (1.00, 1.06)	0.050	0.99 (0.94, 1.04)	0.714	1.05 (1.01, 1.09)	0.008
Model 2						
Light	0.98 (0.94, 1.03)	0.445	0.99 (0.93, 1.05)	0.713	0.98 (0.92, 1.04)	0.427
Moderate	0.96 (0.92, 0.99)	0.013	0.97 (0.92, 1.03)	0.280	0.95 (0.90, 0.99)	0.022
Vigorous	0.88 (0.81, 0.96)	0.005	0.90 (0.80, 1.01)	0.072	0.87 (0.77, 0.99)	0.046
Sedentary time	1.03 (1.00, 1.06)	0.058	0.99 (0.94, 1.05)	0.750	1.05 (1.01, 1.09)	0.009

Adjusted for age, sex, body mass index, alcohol consumption, smoking status, hypertension, hyperlipidemia, diabetes mellitus, and daily-life physical activity.

OR, odds ratio; CI, confidence interval

**Fig. 1.**
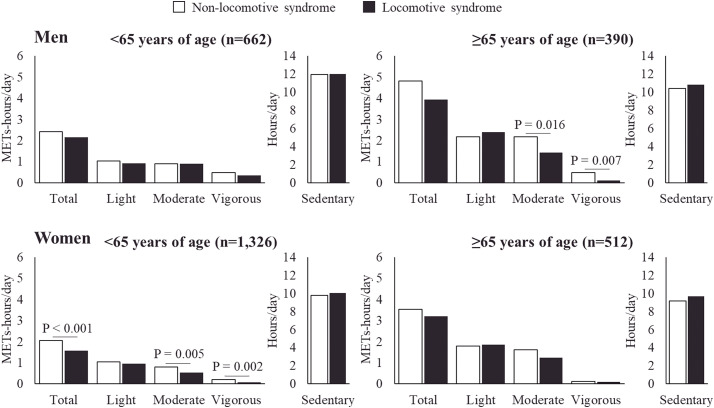
Comparison of leisure-time physical activity and sedentary time with and without locomotive syndrome, stratified by age and sex The top row shows the results for men and the bottom row shows the results for women. The left column shows results for those under 65 years of age, while the right column shows results for those of ≥65 years of age. METs, metabolic equivalents

Figure 1 illustrates a comparison of leisure-time physical activity and sedentary time in individuals with and without LS, stratified by age and sex. The participant distribution was as follows: 662 men aged <65 years, 390 men ≥65 years, 1326 women <65 years, and 512 women ≥65 years. The number of participants with LS based on category was as follows: 249 (37.6%) men aged <65 years, 251 (64.4%) men ≥65 years, 643 (48.5%) women <65 years, and 371 (72.5%) women ≥65 years. No differences in physical activity levels were observed between those with and without LS among men aged <65 years and women ≥65 years of age. By contrast, men aged ≥65 years without LS had significantly higher levels of moderate and vigorous physical activities. Similarly, women aged <65 years without LS had higher levels of total leisure-time physical activity, with particularly significant differences between moderate and vigorous physical activities. No differences in sedentary time were observed between any of the subgroups.

Table 3 displays the results of the logistic regression analysis for LS categorized by age and sex. No significant association was found between leisure-time physical activity, sedentary time, and LS among men aged <65 years and women aged ≥65 years. However, for men aged ≥65 years and women aged <65 years, a distinct pattern was noted. The odds of LS gradually decreased with increasing intensity of physical activity (model 2). Vigorous physical activity was significantly associated with lower odds of LS in men aged ≥65 years. In addition, levels of total leisure-time physical activity were significantly associated with lower odds of LS in women aged <65 years, with moderate and vigorous physical activities showing significant associations. Furthermore, after adjustment for potential confounders, the association between sedentary time and LS was only confirmed in women aged <65 years.

**Table 3.** Association between leisure-time physical activity, sedentary time, and locomotive syndrome, stratified by age and sex

Men	<65 years		≥65 years	
	OR (95%CI)	P	OR (95%CI)	P
Model 1				
Total	0.97 (0.92, 1.02)	0.256	0.96 (0.91, 1.01)	0.094
Sedentary time	0.97 (0.91, 1.04)	0.371	1.04 (0.95, 1.14)	0.424
Model 2				
Light	0.90 (0.81, 1.01)	0.063	1.05 (0.96, 1.16)	0.261
Moderate	1.02 (0.94, 1.10)	0.711	0.94 (0.87, 1.02)	0.115
Vigorous	0.96 (0.85, 1.08)	0.460	0.68 (0.50, 0.91)	0.010
Sedentary time	0.97 (0.91, 1.03)	0.309	1.04 (0.95, 1.15)	0.401
**Women**	**OR (95%CI)**	**P**	**OR (95%CI)**	**P**
Model 1				
Total	0.92 (0.88, 0.96)	<0.001	0.99 (0.94, 1.04)	0.588
Sedentary time	1.05 (1.01, 1.09)	0.027	1.07 (0.98, 1.17)	0.150
Model 2				
Light	0.95 (0.88, 1.02)	0.174	1.01 (0.92, 1.12)	0.785
Moderate	0.92 (0.86, 0.99)	0.021	0.97 (0.90, 1.04)	0.328
Vigorous	0.80 (0.67, 0.96)	0.017	1.04 (0.80, 1.34)	0.795
Sedentary time	1.05 (1.01, 1.09)	0.032	1.07 (0.98, 1.17)	0.151

Adjusted for age, body mass index, alcohol consumption, smoking, hypertension, hyperlipidemia, diabetes mellitus, and daily-life physical activity. The number of participants was as follows: 662 men aged <65 years, 390 men aged ≥65 years, 1,326 women aged <65 years, and 512 women aged ≥65 years.

OR, odds ratio; CI, confidence interval

## Discussion

We investigated the associations among physical activity at different intensities, sedentary time, and LS. This study showed that leisure-time physical activity was associated with LS; a higher physical activity intensity was more strongly associated with LS. In addition, the association between sedentary time and LS was confirmed, but only in women aged <65 years. The associations between physical activity, sedentary time, and LS differed by age and sex.

Our results demonstrated a significant association between physical activity intensity and LS with higher-intensity physical activity showing a stronger association with LS. Systematic reviews^18)^ have consistently highlighted the association between physical activity and a reduced risk of functional limitation and disability in older adults. Moreover, higher-intensity physical activity has been associated with superior performance outcomes, such as walking longer distances or climbing stairs^19)^. We can reasonably assume that higher-intensity physical activity is associated with LS, as risk measures for LS, such as standing on one leg, are more advanced than those encountered in everyday life. While the LS is considered a precursor of sarcopenia^20)^, the importance of high-intensity physical activity in preventing sarcopenia has been emphasized^21)^. Therefore, when prescribing physical activity to prevent or address LS, focusing on the intensity of physical activity is important.

The prevention of LS in women is important because of its higher prevalence than that in men^22,23)^. Our results indicate that the association between LS and physical activity is particularly significant in women under 65 years of age, suggesting that they are prime candidates for interventions that promote physical activity. In women under 65 years of age, total leisure-time physical activity and moderate- and vigorous-intensity physical activities were associated with LS. This suggests that, although high-intensity physical activity is desirable, increasing leisure-time physical activity, regardless of intensity, may also have a preventive effect on LS in this population. This observed association between LS and physical activity in this population could be related to changes in estrogen levels after menopause. Estrogen plays a protective role in muscle function, but its levels decline rapidly following menopause^24)^. Therefore, lifestyle interventions, such as physical activity and nutrition, are essential for preserving muscle function in women beyond the menopausal transition^25)^. Additionally, some studies have suggested that moderate levels of leisure-time physical activity are associated with increased estrogen levels^26)^. Conversely, in women aged ≥65 years, no association was observed between LS and physical activity. Furthermore, the prevalence of LS in this population was as high as 72.5%. These findings suggest the importance of promoting physical activity at an earlier age in women.

The association between LS and physical activity was weaker in men than in women (Table 2). Analysis of the data stratified by age and sex showed no significant association between physical activity and LS in men under 65 years of age, whereas only vigorous physical activity showed a significant association in men ≥65 years of age. As high-intensity physical activity declines with age^27)^, our results indicate the importance of continuing vigorous physical activity at an older age. Previous studies have also reported that exercise habits in middle-aged individuals may prevent LS in old age^4)^. Therefore, our results suggest that promoting high-intensity physical activity before and during old age may help reduce the risk of developing LS in men.

Our results showed that sedentary time was only associated with LS in women aged <65 years. While studies on this association are limited, a study involving 335 participants with a mean age of 44.2 years reported a significant association between sedentary time and LS^7)^. These results suggest that sedentary time may be associated with the prevalence of LS and that initiatives to reduce sedentary time in middle-aged women may help prevent LS. However, the measurement of sedentary time in these studies, including our study, relies on self-reported measures. Regarding the relationship between sedentary time and frailty, the association with sedentary time appears to be weaker for objective measures than for self-reported measures^28)^. Future studies need to investigate the relationship between objectively measured sedentary time and LS.

This study has several limitations. First, as this was a cross-sectional study, causal relationships could not be established. However, existing literature suggests that physical activity plays a role in preventing musculoskeletal health problems, disabilities in activities of daily living, and functional limitations^18)^. Furthermore, regular exercise has been reported to prevent both LS and sarcopenia^4,21)^. Second, in the logistic regression analysis, hard labor and walking were considered as daily-life physical activities, while other activities of daily living were not included. Furthermore, physical activity and sedentary time were assessed using questionnaires rather than objective measures such as accelerometers. Future research should aim to confirm the association between objectively measured physical activity and sedentary time with LS.

## Conclusions

This study investigated the association between physical activity, sedentary time, and LS. Our results confirm the association between physical activity and LS and suggest the importance of engaging people in high-intensity physical activity, especially during middle age. Although an association between sedentary time and LS was noted, this was limited to women aged <65 years. Future longitudinal studies should investigate the associations among objectively measured physical activity, sedentary time, and LS.

## Acknowledgments

We would like to thank Editage for the English language editing.

## Funding

This study was supported by a Grant-in-Aid for Scientific Research on Priority Areas of Cancer (Grant No. 17015018) and on Innovative Areas (Grant No. 221S0001), as well as by JSPS KAKENHI (Grant Nos. 16H06277, 22H04923, 22K10588, 22K11190, and 24K14235) from the Ministry of Education, Culture, Sports, Science and Technology of Japan.

## Conflicts of Interest

The authors have no conflict of interest to disclose.
